# Depressive symptoms in children and adolescents with epilepsy and primary headache: a cross-sectional observational study

**DOI:** 10.3389/fneur.2024.1395003

**Published:** 2024-06-25

**Authors:** Grazia Maria Giovanna Pastorino, Miriam Olivieri, Andrea Viggiano, Rosaria Meccariello, Michele Roccella, Lucia Parisi, Emanuele Cerulli Irelli, Carlo Di Bonaventura, Alessandro Orsini, Francesca F. Operto

**Affiliations:** ^1^Child and Adolescent Neuropsychiatry Unit, Department of Medicine, Surgery, and Dentistry, University of Salerno, Salerno, Italy; ^2^Department of Medicine, Surgery and Dentistry, University of Salerno, Baronissi, Italy; ^3^Department of Movement and Well-Being Sciences, Parthenope University of Naples, Naples, Italy; ^4^Department of Psychology, Educational Science and Human Movement, University of Palermo, Palermo, Italy; ^5^Department of Human Neurosciences, Sapienza University, Rome, Italy; ^6^Pediatric Neurology, Azienda Ospedaliero-Universitaria Pisana, Pisa University Hospital, Pisa, Italy; ^7^Department of Science of Health, School of Medicine, University Magna Graecia of Catanzaro, Catanzaro, Italy

**Keywords:** primary headache, epilepsy, depressive symptoms, parental stress, children, adolescents, CDI-2

## Abstract

**Background:**

The primary aims of our cross-sectional observational study were: (i) to determine the prevalence of depressive symptoms in children and adolescents with epilepsy compared to controls and (ii) to explore the difference in depressive symptoms in patients with epilepsy only and those with epilepsy and primary headache as a comorbidity. The secondary objective was to explore parental stress levels.

**Methods:**

68 pediatric patients aged 6–18 years (44 with epilepsy only and 24 with epilepsy and headache) and 50 controls were recruited. Depressive profile and parental stress were assessed using Children's Depression Inventory, Second Edition (CDI-2) and Parenting Stress Index-Short Form (PSI-SF).

**Results:**

The group with epilepsy showed significantly high depressive symptoms and parental stress compared to controls. The patients with headache in comorbidity experienced more depressive symptoms than those with epilepsy only.

**Conclusion:**

Depressive symptoms are more prevalent in patients who have comorbid epilepsy and primary headache; therefore, the neurological/psychological mechanisms underlying this condition should be further investigated. The simultaneous presence of epilepsy, headache and depressive symptoms impacts the quality of life of patients and their parents, increasing parental stress and family management.

## 1 Introduction

Epilepsy affects 0.5–1% of children worldwide and is one of the most common neurological disorders (1). It is also associated with an increased risk of developing various pathologies, especially psychiatric. The International League Against Epilepsy (ILAE) has estimated that 35–50% of children with epilepsy experience neuropsychiatric comorbidities (2). This can include a range of conditions such as depression, anxiety, attention-deficit/hyperactivity disorder (ADHD) and autism spectrum disorder (ASD) (3). For centuries, the bidirectional relationship between epilepsy and neuropsychiatric comorbidities has been documented, but the mechanisms underlying this correlation are not yet fully understood. However, several factors have been suggested to play a role in this relationship, including genetic and psychosocial background, epileptic activity and neurochemical and electrophysiologic changes related to the seizure disorder and antiepileptic treatment (4, 5). The presence of comorbid mental health conditions negatively impacts the quality of life and functioning of people with epilepsy and their caregivers (6). It is also clinically significant, as it complicates the management of epilepsy.

Depressive disorders are the most common psychiatric comorbidity associated with epilepsy in children and adolescents. In several clinic-based studies, the prevalence of depression in children and adolescent with epilepsy ranges from 12.7% to 36.5% (7). The symptomatology of mood disorders in patients with epilepsy is varied and can be challenging to identify. They can manifest as clusters of depressive symptoms, including sadness, crying attacks, feelings of anhedonia, and irritability. Alternatively, they may manifest as well-characterized disorders that meet the diagnostic criteria of the Diagnostic and Statistical Manual of Mental Disorders—Fifth Edition (DSM-V). About 50% of patients experience an interictal dysphoric disorder (IDD). It is not yet known whether IDD is a distinctive disease, or if it is a manifestation of mood disorders in epilepsy (8). The term IDD was introduced by Blumer to describe a pleomorphic affective disorder that emerges in epilepsy. The characteristics of IDD are labile depressive symptoms, such as depressive mood, anergy, pain, insomnia, and labile affective symptoms (fear, anxiety) and specific features, including euphoria and paroxysmal irritability. These symptoms appear intermittently, without external triggers, and typically last from a few hours up to 2 days, with a rapid onset and resolution (9). In a recent study conducted in Barcelona, 98 adults with drug- resistant epilepsy were diagnosed with IDD and 70 of them had also psychiatric disorder, according to Structured Clinical Interview for DSM-IV (SCID-IV) (10). These subjects had a different psychopathological profile and quality of life (QoL) scores compared to subjects with drug-resistant epilepsy without IDD. In fact, the authors found a statistically significant association between IDD and obsessive–compulsive, borderline, and depressive personality disorder, and significantly lower scores in all Quality of Life in Epilepsy (QOLIE-31) items except for “medication effects” in subjects with IDD compared with those without IDD. This study suggests that psychiatric symptomatology, when occurs in people with epilepsy, cannot be included in the standardized classification systems and a better and deeper understanding of psychopathology in these subjects through psychometric scales could help improve clinical practice in these specific patients.

Headache is another neurological condition that frequently occurs in the pediatric population, affecting one-third to one-half of children and adolescents. Headaches are classified into primary headache disorders, like migraine, and the secondary headache disorders, with an underlying condition. Migraine is the most common type of headache that occurs in children with an overall prevalence of about 7–8%. This prevalence increases in adolescence. In fact, the literature shows a prevalence of episodic migraine at approximately 2–5% in preschool children, 10% in school-aged children, and 20–30% in adolescents (11). During childhood, boys and girls are equally affected, whereas in adolescence, women are more affected. There are different characteristics in pediatric and adult patients with migraine. Headaches in children are generally shorter compared to adults and are reported bilaterally before adolescence. Furthermore, children may have difficulties verbalizing their symptoms and the pain often needs to be assumed from their behaviors (12). The bidirectional link between epilepsy and migraine is still debated. Epidemiological studies demonstrate that epilepsy is more frequent in subjects with migraine than in the general population. Similarly, migraine is also more frequent in people with epilepsy (13). Although the two conditions differ, they share some similarities. In fact, both disorders are distinguished by transient paroxysmal episodes of altered brain function with a clinical, pathophysiological, and therapeutic overlap (14). They share common substrates, such as alterations in neuronal transmission or genetic basis and several triggers, such as sleep deprivation, bright flashing lights, and stress.

The coexistence of primary headache, depressive symptoms, and epilepsy in both pediatric and adult populations is indeed a fascinating area of study. The simultaneous presence of these conditions suggests a common substrate, not yet well understood. However, these conditions affect patients' wellbeing and treatment outcomes.

The primary aim of our cross-sectional observational study was (i) to determine the difference between the prevalence of depressive symptoms in the study group (patients with epilepsy only and patients with epilepsy and primary headache) compared to control group and (ii) to research the difference in depressive symptoms in the subgroup with only epilepsy and in those with epilepsy and headache in comorbidity. The secondary objective was to explore stress levels in the parents of children with epilepsy and primary headache, compared to those in the control group.

## 2 Materials and methods

### 2.1 Participants

Sixty-eight pediatric patients aged 6–18 years (44 with epilepsy only and 24 with epilepsy in comorbidity with primary headache) were recruited from the Child Neuropsychiatry Unit of the University of Salerno (Italy) between January 2019 and May 2023.

The inclusion criteria were diagnosis of epilepsy; diagnosis of primary headache; age between 6 and 18 years; and availability to complete our questionnaires. The exclusion criteria were presence of severe neurological or psychiatric pathologies (particularly syndromes or severe intellectual disabilities) and poor parental compliance.

The diagnosis of epilepsy was made by a child neuropsychiatrist with experience in the treatment of epilepsy, based on clinical history, clinical manifestations of seizures, and video-electroencephalogram (EEG) characteristics, according with the International League Against Epilepsy classification (15). Primary headache was diagnosed based on clinical history, and children's behavior, according to the criteria of the International Classification of Headache Disorders, Third Edition (ICHD-III Beta version) of the International Headache Society (IHS) (16).

The controls were recruited from the general population participating in a screening program for learning disabilities carried out in collaboration with the University of Salerno. The recruited subjects were tested by two child neuropsychiatrists and any form of epilepsy and headache had previously been excluded through medical history, neurological examination, and EEG when necessary.

The characteristics of the study group and control group are summarized in Table 1.

**Table 1:** Main sample characteristics.

	**Study group (*N* = 68)**	**Control group (*N* = 50)**
**Sex (** ***N*** **; %)**		
Men	41 (60%)	30 (60%)
Women	27 (40%)	20 (40%)
**Age (yrs)**		
Range	6–18	6–18
Mean	12.76 ± 3.32	12.42 ± 3.10
**Diagnosis (** ***N*** **; %)**		
Epilepsy	44 (65%)	
Epilepsy + primary headache	24 (35%)	
**Epilepsy type (** ***N*** **; %)**		
Focal	40 (59%)	
Generalized	21 (31%)	
Unknown	7 (10%)	
Drug resistance (*N*; %)	17 (25%)	
**Primary Headache (** ***N*** **; %)**		
Type cluster-headache	1 (4%)	
Migraine	7 (29%)	
With aura	2 (8%)	
Without aura	5 (21%)	
Tension-type headache	16 (67%)	
**Frequency of headache attacks (** ***N*** **; %)**		
Daily	1 (4%)	
Multi-weekly	4 (17%)	
Weekly	12 (50%)	
Monthly	6 (25%)	
**Pain intensity (** ***N*** **; %)**		
Mild	6 (25%)	
Moderate	13 (54%)	
Severe	5 (21%)	
Headache onset (mean in years)	12.04 ± 2.61	
Headache duration (mean in years)	1.46 ±1.32	

Depressive profile and parental stress were assessed using Children's Depression Inventory, Second Edition (CDI-2) Self Report and Parents Form (17) and Parenting Stress Index—Short Form (PSI-SF) (18). Both questionnaires were administered in the validated and standardized Italian version, for the Italian population.

All the data were collected and analyzed by a team composed of four child psychologists, specifically trained for the administration of the neuropsychological tests, and two child neuropsychiatrist experts in epilepsy and other neurological/psychiatric disorders.

All the participants were informed of the objectives and methods of study, and the parents signed a written informed consent. The study complied with the rules of good clinical practice of the Declaration of Helsinki and approved by the Campania Sud Ethics Committee.

### 2.2 Children's depression inventory, second edition (CDI-2)

CDI-2 is a rating scale that assesses depressive symptoms in children and young people aged 7–17 years (17). The CDI-2-Self Report (CDI-2SR) consists of 28 items divided into two mains scale and subscales. The first main scale, titled Emotional Problems scale, includes Negative Mood/Physical Symptoms and Negative Self-Esteem, and component items evaluate symptoms of distress, such as sadness, guilt, self-loathing, and anomalies in sleep patterns, eating habits, and energy levels. The second main scale, titled Functional Problems Scale, includes Ineffectiveness and Interpersonal Problems, and component items indicate inhibited social relationships, such as peer and family relationships and maladjustment in school. The Likert scale, ranging from 0 (no symptoms) to 2 (definite symptoms), is used.

The 17 items on Children Depression Inventory 2 Parent (CDI-2P) correspond to appropriately rephrased items on the self-report version. Each item is provided by four choices, corresponding to four levels of symptoms: 0 (not at all), 1 (some of the time), 2 (often), or 3 (most of the time). The CDI-2P is designed to be administered by parents or alternative caregivers. The scores of the individual scales add up to obtain the total raw score and then converted to T-score. Therefore, higher T-scores (≥60) reflect a higher incidence of depressive symptoms and are in the “pathological range,” in which it is possible to distinguish between medium-high (60–64), high (65–69), and very high (>70) values.

### 2.3 Parenting stress index- short form (PSI-SF)

Parenting Stress Index is a standardized scale that measures stress in parent–child interactions and returns scores of parenting stress across four domains (18):

—Parental distress (PD): level of parental distress arising from factors related to the parental role;

—Dysfunctional parent–child interaction (PCDI): parental perception of a child who does not respond to family expectations;

—Difficult child (DC): parental stress developing from some specific characteristics of the child;

—Total stress (TS): stress index related to the parental role, obtained by the sum of the other scores.

PSI-SF comprises 36 items, with each item graded on a five-point Likert scale; scores ≥ 85 are in the “pathological range” and those <85 are in the “normal range.”

### 2.4 Statistical analysis

All clinical variables were subjected to statistical analysis. Categorical variables were presented using counts and percentages, while continuous variables were described as mean and standard deviation (SD).

To verify the data distribution, we preliminarily carried out the Kolmogorov–Smirnov normality test. Given the non-normal distribution of the data, we chose to employ a non-parametric test. To evaluate the differences in proportion, we used chi-square test. To evaluate significant differences in mean scores, we performed the Kruskal–Wallis test and *post-hoc* analysis by Mann–Whitney U test for independent sample; *p* ≤ 0.05 was considered statistically significant. Statistical Package for Social Science (SPSS) version 23.0 (IBM Corporation, Armonk, NY, USA)was used for statistical analysis.

## 3 Results

### 3.1 Comparison of main characteristics between group with epilepsy only and group with epilepsy + primary headache

The two groups (epilepsy only and epilepsy + primary headache) were comparable with regard to main clinical and demographic characteristics. The results are summarized in Table 2. No statistically significant differences were detected between the two groups and clinical and demographic characteristics, such as age, sex, seizure type, and seizure frequency, and Antiseizure Medications (ASM) numbers.

**Table 2:** Statistical comparison between group with epilepsy only and epilepsy + primary headache.

	**Epilepsy only (*N* = 44)**	**Epilepsy + primary headache (*N* = 24)**	**Statistical analysis**
Age (mean)	12.36 ± 3.18 years	13.5 ± 12.76 years	U Mann–Whitney p = 0.209 Z = −1.256
Sex	Men = 26 (59%) Women = 18 (41%)	MeN = 15 (62.5%) WomeN = 9 (37.5%)	Chi-square p = 0.783 X^2^= 0.0754
Seizure type	Focal = 25 Generalized = 15 Unknown = 4	Focal= 15 Generalized=6 Unknown = 3	Chi-square p = 0.713 X^2^ = 0.6761
Seizure frequency	1.2 ± 2.11 (months)	1.88 ± 2.72 (months)	U Mann–Whitney p = 0.237 Z = −1.183
ASM numbers (mean)	1.32 ± 0.56	1.63 ± 0.77	U Mann–Whitney P = 0.087 Z = −1.710

ASMs, antiseizure medications.

### 3.2 Results from CDI-2 self report and CDI-2 parents

The comparison between mean CDI-2 self-report and CDI-2 Parent scores between the three groups, carried out using the Kruskal–Wallis test, showed statistically significant differences in all scales analyzed (Table 3). In particular, the children and adolescents with epilepsy report significantly medium-high levels in all the subscales, compared to those in the control group. The results obtained by parent-reported questionnaire showed significantly high levels than controls in all the subscales. The *post-hoc* analysis is performed by Mann–Whitney U test and is summarized in Table 4. The comparison between the mean scores of CDI-2 Self Report and CDI-2 Parents in patients with epilepsy vs. patients with epilepsy and primary headache showed that patients with headache obtained significantly high scores in all the subscales analyzed, except for Emotional Problems scale—CDI-2 Parents.

**Table 3:** Comparison between CDI-2 scores in the group with epilepsy only, epilepsy + primary headache and controls.

**CDI-2 self report**				
	**Epilepsy only (mean** ±**SD)**	**Epilepsy** + **primary headache (mean** ±**SD)**	**ControlGroup (mean** ±**SD)**	**Kruskal–Wallis test**
Total score	62.54 ± 10.73	48.98 ± 7.10	43.31 ± 4.31	**p < 0.001 X**^**2**^ **= 56.942**
Mood	61.58 ± 10.02	50.11 ± 7.90	44.01 ± 5.73	**p < 0.001 X**^**2**^ **= 50.505**
Self-esteem	60.88 ± 9.24	48.02 ± 8.67	42.72 ± 5.04	**p < 0.001 X**^**2**^ **= 55.550**
Inefficacy	60.00 ± 9.56	48.93 ± 7.86	44.28 ± 5.67	**p < 0.001 X**^**2**^ **= 44.377**
Interpersonal problems	61.21 ± 10.49	50.46 ± 8.34	44.31 ± 5.31	**p < 0.001 X**^**2**^ **= 48.359**
Emotional problems	62.13 ± 10.82	48.32 ± 7.52	43.54 ± 5.01	**p < 0.001 X**^**2**^ **= 52.910**
Functional problems	59.33 ± 11.57	50.59 ± 6.78	45.09 ± 5.43	**p < 0.001 X**^**2**^ **= 40.253**
**CDI-2 parents**				
Total score	63.79 ± 16.25	54.32 ± 11.81	45.22 ± 12.61	**p < 0.001 X**^**2**^ **= 33.353**
Emotional problems	60.92 ± 16.88	52.80 ± 12.49	45.12 ± 13.52	**p < 0.001 X**^**2**^ **= 20.519**
Functional problems	65.79 ± 17.22	54.05 ± 11.32	48.25 ± 13.60	**p < 0.001 X**^**2**^ **= 29.104**

p ≤ 0.05 are in bold.

**Table 4:** *Post-hoc* analysis of CDI-2 self-report and CDI-2 parents (Mann–Whitney U test).

**CDI-2 self report**			
	**Epilepsy only vs. controls**	**Epilepsy** + **headache vs. controls**	**Epilepsy only vs. epilepsy** + **headache**
Total score	**p < 0.001 Z = −4.781**	**p < 0.001 Z = −6.587**	**p < 0.001 Z = −4.671**
Mood	**p < 0.001 Z = −4.465**	**p < 0.001 Z = −6.363**	**p < 0.001 Z = −4.161**
Self-esteem	**p < 0.001 Z = −4.533**	**p < 0.001 Z = −6.728**	**p < 0.001 Z = −4.508**
Inefficacy	**p < 0.001 Z = −3.537**	**p < 0.001 Z = −6.158**	**p < 0.001 Z = −4.238**
Interpersonal problems	**p < 0.001 Z = −4.478**	**p < 0.001 Z = −6.121**	**p < 0.001 Z = −3.845**
Emotional problems	**p < 0.001 Z = −3.638**	**p < 0.001 Z = −6.770**	**p < 0.001 Z = −4.967**
Functional problems	**p < 0.001 Z = −4.038**	**p < 0.001 Z = −5.667**	**p = 0.001 Z = −3.437**
**CDI-2 parents**			
Total score	**p < 0.001 Z = −4.387**	**p < 0.001 Z = −4.693**	**p = 0.014 Z = −2.453**
Emotional problems	**p = 001 Z = −3.212**	**p < 0.001 Z = −3.901**	p = 0.057 Z = −1.902
Functional problems	**p < 0.001 Z = −3.782**	**p < 0.001 Z = −4.530**	**p = 0.005 Z = −2.783**

p ≤ 0.05 are in bold.

### 3.3 Results from PSI-SF

The comparison between mean PSI-SF scores between the three groups, performed by the Kruskal–Wallis test, showed statistically significant differences in all scales analyzed (Table 5). The *post-hoc* analysis is performed by Mann–Whitney U test and is summarized in Table 6. In particular, the comparison between the mean scores of PSI in patients with epilepsy vs. patients with epilepsy and primary headache showed that patients with headache obtained significantly high scores in the Parental Distress scale. No statistically significant differences were detected in the other subscales (Table 6).

**Table 5:** Comparison between PSI scores in group with epilepsy only, epilepsy + primary headache and controls.

	**Epilepsy only (mean ±SD)**	**Epilepsy + primary headache (mean ±SD)**	**Control group (mean ± SD)**	**Kruskal–Wallis test**
Parental distress	72.95 ± 31.72	55.00 ± 30.04	31.10 ± 17.14	**p < 0.001 X**^**2**^ **= 42.286**
Parent-child Difficult interaction	79.20 ± 22.87	63.54 ± 21.89	40.51 ± 19.55	**p < 0.001 X**^**2**^ **= 58.238**
Difficult child	77.50 ± 27.50	69.79 ± 31.40	35.29 ± 16.07	**p < 0.001 X**^**2**^ **= 51.494**
Total stress	77.50 ± 28.66	66.70 ± 30.10	34.41 ± 16.70	**p < 0.001 X**^**2**^ **= 51.611**

p ≤ 0.05 are in bold.

**Table 6:** *Post-hoc* analysis of PSI-SF (Mann–Whitney U test).

	**Epilepsy only vs. controls**	**Epilepsy + headache vs. controls**	**Epilepsy only vs. epilepsy + headache**
Parental distress	**p < 0.001 Z = −3.614**	**p < 0.001 Z = −6.174**	**p = 0.027 Z = −2.216**
Parent-child difficult Interaction	**p < 0.001 Z = −5.300**	**p < 0.001 Z = −6.867**	p = 0.172 Z = −1.365
Difficult child	**p < 0.001 Z = −4.329**	**p < 0.001 Z = −6.801**	p = 0.232 Z = −1.196
Total stress	**p < 0.001 Z = −4.321**	**p < 0.001 Z = −6.770**	p = 0.084 Z = −1.726

p value ≤ 0.05 are in bold.

## 4 Discussion

Several studies have reported that, in children and youth with epilepsy (YWE), psychiatric comorbidities, such as anxiety, depression, and attention-deficit/hyperactivity disorder (ADHD) are more prevalent compared to the general population.

The possible risk factors among these conditions are not yet known. According to a recent meta-analysis (19), the prevalence of anxiety and depression in YWE is 18.9% and 13.5%, respectively. However, the examined studies showed significant heterogeneity between the samples, and the authors did not find a significant correlation with age, sex, polytherapy, duration of epilepsy, and type of diagnosis. In samples of YWE compared with healthy controls, significantly higher anxiety and depressive symptomatology was reported. In these cases, the authors found that age and duration of epilepsy were significant moderators, unlike sex, polytherapy, and age of epilepsy onset.

In a cohort study of 80 children (6–13 years) with epilepsy, Shehata et al. (20) found that 37.5% of them had depressive symptoms. The highest predictor of depression was sleep disturbance, detected in 90% of cases, followed by appetite disturbance in 86.6% of the cases, whereas delusions and hallucinations were detected in only 10% of the cases. The study also found that the duration of epilepsy more than 3 years and more frequent seizures were significantly higher in cases with depressive symptoms.

Similar results were found in a recent study, conducted in Oman, with 75 children with epilepsy, aged 6–12 years (21). The authors found that younger children had difficulty expressing their mood and sadness, reporting fewer depressive symptoms, which were represented by irritability, anger, and sleep disturbances. This study also found that children with focal epilepsy are more likely to experience depressive symptoms.

Migraine is the second most prevalent and disabling disease worldwide, especially in developmental age population. There is strong evidence in the literature of the increased risk of anxiety and depression in children and adolescents with migraine. A recent systematic review and meta-analysis of 80 observational studies (22) found that children and adolescents with migraine experience more depressive and anxiety symptoms and disorders than their peers without migraine. Screening for these symptoms and disorders may be helpful in clinical practice, as they can significantly affect the overall wellbeing of individuals.

The main aim of our study was to estimate the prevalence of depressive symptoms in children and adolescents with epilepsy and migraine, using CDI-2 (Self-Report form and Parent form), a standardized and validated questionnaire, which evaluates depressive symptoms in children and young people. The current study is the first, to our knowledge, to use both CDI-2 form for patients and their parents to detect the depressive symptoms in a population with epilepsy.

The results obtained by this patient-self-reported questionnaire showed significantly medium-high levels in all the subscales compared to those in the control group. These findings are consistent with the current literature. Research conducted by Guifoyle et al. in 311 YWE between 7 and 17 years of age found depression in 23% of them, with 14% endorsing suicidal ideation. The authors also found that depression significantly varied based with age, antiepileptic drug, and insurance status (23).

The results obtained by parent-reported questionnaire showed significantly high levels than controls in all the subscales. This slight difference between the mean score in the Self Report form and the Parent Form could be due to the different perceptions of health between patients and their parents or other biases, such as honesty, social desirability, and introspective ability.

These findings suggest that CDI-2 SR should be included in combination with other validated tests specific for the population with epilepsy. Psychiatric or psychological assessments structured or semi-structured interviews, and self-report screening tools can be used to detect depression or depressive symptoms (24). Several depression screening tools have been developed for the general population, including the Beck Depression Inventory (BDI) (25), the Hospital and Anxiety and Depression Scale (HADS) (26), and the Hamilton Rating Scale for Depression (HRSD) (27), and have been validated by using gold and/or reference standards various screening tools such as Structured Clinical Interview for DSM-IV-TR Axis I Disorders (SCID-I) (28) or Mini International Neuropsychiatric Interview (MINI) (29). The Neurological Disorders Depression Inventory for Epilepsy for Youth (NDDI-E-Y) (30) is a validated self-report screening tool designed to assess depressive symptoms in pediatric epilepsy patients aged 12–17 years, allowing for early intervention and support. Diagnosis depressive symptoms or disorders in children and adolescents can be challenging because of nuanced and sometimes vague clinical presentation. Additionally, children may be unable to express their emotions verbally. For these reasons, the use of these diagnostic assessment tools should be accompanied by a thorough clinical investigation and a careful psychic examination. In addition, the environment (parental type, dynamics habitual pedagogical attitudes, socio-economic status), the presence of stressful events, and the cognitive and adaptive profile of patients should be included in the clinical evaluation.

Due its comorbidities and complex management, epilepsy is a disabling condition, that negatively affects family wellbeing. For this reason, we also measured the stress level in parents of children and adolescents with epilepsy to determine if and how parental stress is linked to the condition of their children. According to previous literature data (31–33) and using PSI as a psychodiagnostics tool, we found statistically significant levels in terms of parenting stress in the study group compared to controls.

Analyzing the study group in detail, we divided patients with epilepsy (*N* = 44) and patients who had a diagnosis of epilepsy and primary headache in comorbidity (*N* = 24) and compared the results obtained from the questionnaires administered to them.

The comparison between the mean scores of CDI-2 Self Report and CDI-2 Parents in patients with epilepsy vs. patients with epilepsy and primary headache showed that patients with headache obtained significantly high scores in all the subscales analyzed, except for Emotional Problems scale—CDI-2 Parents. Similarly, the comparison between the mean scores of PSI in patients with epilepsy vs. patients with epilepsy and primary headache showed that patients with headache showed significantly high scores in the Parental Distress scale. No statistically significant differences were detected in the other subscales.

These findings are in line with the literature and suggest that children and adolescents who have both epilepsy and primary headache are more at risk of developing depressive symptoms or disorders. Our previous research, using Child Behavior Checklist (CBCL) questionnaire (34) found a significant difference in affective problems and anxious and somatic complaints between the group with headache and the control group. These findings validate the existence of an emotional component associated with headaches and highlight that headache is a multifaceted biomedical condition, wherein psychological factors may contribute to its persistence or reactivity.

Nevertheless, these two comorbid disorders of epilepsy have been correlated with a worse quality of life (35) and a significant clinical impact. Indeed, there remain significant unanswered questions: the impact of these comorbidities and their treatment on the severity and course of the seizure disorder (36).

Some limitations inherent in this study should be reported. The first limitation is the small sample size, which may be justified by the general difficulty in recruiting healthy subjects; the generalization of findings and analyses for all the subtypes of epilepsy and primary headache were not allowed due to the relatively small study sample. For this reason, we have not analyzed and compared the features of epilepsy and primary headache, such as frequency, intensity, and duration. There may be a possibility that these characteristics (uncontrolled seizures, increased frequency, or duration) may affect the complexity of the clinical picture. The second limitation is related to the stress and depressive symptoms assessment method used. Although PSI and CDI-2 have good psychometric properties, they are subjective self-related measures that could lead to a reprehensible prejudice. However, to our knowledge, this is the first study using both CDI-2 forms to detect depressive symptoms in children and adolescents with epilepsy and primary headache, confirming the complex relations among common neurologic and psychiatric comorbidities of epilepsy and the importance of an early detection and intervention. Further research probing the role of these conditions in the course and treatment of the seizure disorder is needed.

## 5 Conclusion

Our study highlighted the high occurrence of depressive symptoms in children and adolescents with epilepsy. Depressive symptoms are more prevalent in patients who have comorbid epilepsy and primary headache; therefore, the neurological/psychological mechanisms underlying this condition should be further investigated.

The simultaneous presence of epilepsy, headache, and depressive symptoms impacts the quality of life of patients and their parents, increasing parental stress and family management.

## Data availability statement

The raw data supporting the conclusions of this article will be made available by the authors, without undue reservation.

## Ethics statement

The studies involving humans were approved by Campania Sud Ethics Committee. The studies were conducted in accordance with the local legislation and institutional requirements. Written informed consent for participation in this study was provided by the participants' legal guardians/next of kin. Written informed consent was obtained from the individual(s) for the publication of any potentially identifiable images or data included in this article.

## Author contributions

GP: Conceptualization, Formal analysis, Writing – original draft, Writing – review & editing, Data curation, Methodology. MO: Data curation, Writing – original draft, Writing – review & editing, Methodology. AV: Formal analysis, Writing – review & editing. RM: Methodology, Writing – review & editing. MR: Conceptualization, Writing – review & editing. LP: Writing – review & editing. EC: Writing – review & editing. CD: Writing – review & editing. AO: Writing – review & editing. FO: Conceptualization, Supervision, Writing – review & editing.
